# Crop Leaf Phenotypic Parameter Measurement Based on the RKM-D Point Cloud Method

**DOI:** 10.3390/s24061998

**Published:** 2024-03-21

**Authors:** Weiyi Mu, Yuanxin Li, Mingjiang Deng, Ning Han, Xin Guo

**Affiliations:** 1 School of Water Resources and Hydropower, Xi’an University of Technology, Xi’an 710054, China; yuanxin0325@163.com (Y.L.); xjdmj@163.com (M.D.); hn15027813753@163.com (N.H.); guoxin131127@163.com (X.G.); 2 Department of Water Resources of Xinjiang Uygur Autonomous Region, Urumqi 830099, China

**Keywords:** point cloud, point cloud processing, phenotype parameter, method of measurement

## Abstract

Crop leaf length, perimeter, and area serve as vital phenotypic indicators of crop growth status, the measurement of which is important for crop monitoring and yield estimation. However, processing a leaf point cloud is often challenging due to cluttered, fluctuating, and uncertain points, which culminate in inaccurate measurements of leaf phenotypic parameters. To tackle this issue, the RKM-D point cloud method for measuring leaf phenotypic parameters is proposed, which is based on the fusion of improved Random Sample Consensus with a ground point removal (R) algorithm, the K-means clustering (K) algorithm, the Moving Least Squares (M) method, and the Euclidean distance (D) algorithm. Pepper leaves were obtained from three growth periods on the 14th, 28th, and 42nd days as experimental subjects, and a stereo camera was employed to capture point clouds. The experimental results reveal that the RKM-D point cloud method delivers high precision in measuring leaf phenotypic parameters. (i) For leaf length, the coefficient of determination (R^2^) surpasses 0.81, the mean absolute error (MAE) is less than 3.50 mm, the mean relative error (MRE) is less than 5.93%, and the root mean square error (RMSE) is less than 3.73 mm. (ii) For leaf perimeter, the R^2^ surpasses 0.82, the MAE is less than 7.30 mm, the MRE is less than 4.50%, and the RMSE is less than 8.37 mm. (iii) For leaf area, the R^2^ surpasses 0.97, the MAE is less than 64.66 mm^2^, the MRE is less than 4.96%, and the RMSE is less than 73.06 mm^2^. The results show that the proposed RKM-D point cloud method offers a robust solution for the precise measurement of crop leaf phenotypic parameters.

## 1. Introduction

Phenotypic parameters such as leaf length, perimeter, and area are crucial indicators of crop growth dynamics [1]. The leaf length reflects the crop growth status and guides irrigation strategies, while the leaf perimeter and area correlate with photosynthetic efficiency and yield [2,3,4,5]. Across growth stages, leaf phenotypic parameters exhibit distinct trends. In the initial weeks after germination, leaves experience rapid growth, followed by a maturation phase with slower growth rates. Eventually, the leaf length, perimeter, and area reach their maximum values before potential wilting occurs [6].

Variations in leaf phenotypic parameters across growth stages are essential for understanding crop growth patterns, management strategies, and yield enhancement opportunities.

(1) Point cloud processing method

Three-dimensional (3D) point clouds facilitate the non-invasive measurement of leaf phenotypic parameters, offering advantages over manual and two-dimensional (2D) methods [7,8,9,10,11]. However, original 3D plant point clouds often include irrelevant ground and noise points, complicating accurate parameter measurements [12].

Ground removal, segmentation, and smoothing are essential for improving measurement precision [12]. Traditional ground point cloud removal methods, such as statistical and bilateral filtering, may encounter challenges due to the unpredictable nature of noise points in pepper growth environments [13,14].

Segmentation methods such as the Mean Shift and the K-means algorithm address spatial distribution and geometric properties. While the Mean Shift excels in handling clusters of varying shapes, K-means demonstrates a rapid convergence speed [15,16].

Smoothing techniques such as filtering and Moving Least Squares (MLS) aim to enhance the point cloud quality. Combining filtering and MLS methods can effectively mitigate noise and preserve target features [17,18].

Overall, addressing challenges in point cloud processing, segmentation, and smoothing is crucial for robust and adaptive applications.

(2) Methods for measuring phenotypic parameters

Beyond point cloud processing, selecting suitable methods for measuring crop phenotypic parameters is crucial for robust analysis.

While traditional manual measurements are simple and do not require complex equipment, their processes are susceptible to human error and are limited in scope. In contrast, methods based on image processing offer efficiency and scalability for large-scale measurements; however, they demand strict environmental conditions, with lighting and capture angles potentially affecting the measurement accuracy. Recently, there has been an adoption of deep learning for measuring leaf phenotypic parameters, which exhibits strong adaptability to complex scenarios. Nonetheless, it comes with notable drawbacks, requiring extensive annotated data and long model training cycles and posing potential issues with generalization. Methods utilizing three-dimensional point clouds for phenotypic parameter measurements are also prevalent, offering richer spatial information to capture the three-dimensional structure of leaves; however, their point cloud processing procedures are intricate.

To be specific, the Oriented Bounding Box (OBB) method is commonly used for length, width, and height measurements, but it has limitations with small and curved targets such as leaves [19,20]. UAV remote sensing and structured light projection methods offer alternatives for three-dimensional structural information extraction but have limitations such as field of view constraints and sensitivity to environmental factors.

In consideration of the challenges posed by complex three-dimensional pepper leaf point cloud processing and less precise measurements of phenotypic parameters, in this paper, a novel leaf phenotypic parameter measurement method based on point clouds is proposed, termed RKM-D, which integrates point cloud spatial properties, color characteristics, and Euclidean distance. Tailored point cloud processing methods and leaf phenotypic parameter measurement techniques are proposed to address specific requirements. Validation and analysis against manually measured results confirm the effectiveness of the proposed approach, offering a robust theoretical foundation for related research and applications.

## 2. Materials and Methods

### 2.1. Experimental Design

The experiment focused on three pepper plants, selected as experimental subjects, across three growth stages: the 14th, 28th, and 42nd days. Seventy-two point clouds were collected for each leaf. The LenaCV stereo camera system (Wuhan Lina machine Vision Technology Co., Ltd., Wuhan, China) was employed to comprehensively capture the raw point cloud data.

**Figure 1:**
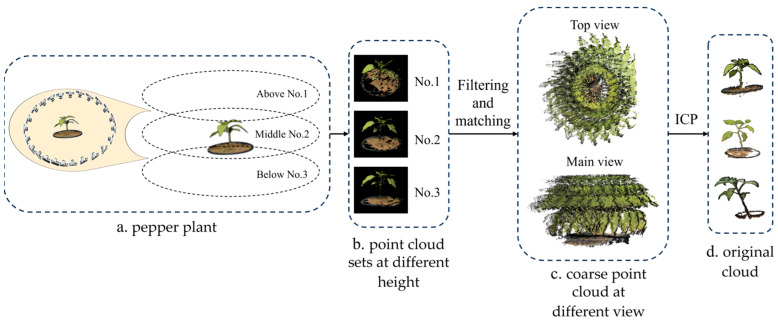
Overview of data acquisition.

As shown in Figure 1, for every pepper plant, the point cloud data were collected at three heights: above (No. 1), middle (No. 2), and below (No. 3). During the collection process, the camera rotated relative to the central axis of the pepper plant, collecting a point cloud dataset every 15 degrees in the circumference of the pepper plant. Therefore, approximately 72 point cloud sets were collected for each pepper plant in each period.

### 2.2. Point Cloud Acquisition Method

As shown in Figure 1, after the collection of each point cloud set, all the point cloud sets were formed into a coarse pepper point cloud using a combination of the wavelet layered filtering method and the homogeneous feature matching method. The denoising of point clouds was achieved through the utilization of wavelet transforms. Specifically, the point cloud data were transformed to generate a series of wavelet coefficients, which were then used to determine the validity of the point cloud [21]. Subsequently, a uniform feature matching method was employed to identify similar features within the point cloud, thereby aggregating multiple point clouds into a rough point cloud, serving as the preliminary registration result [22]. Finally, the original pepper point cloud was obtained by using the ICP registration method [23].

### 2.3. Point Cloud Processing Method

#### 2.3.1. RANSAC-B-Based Ground Points Removal

For the removal of the ground cloud points of the original pepper point cloud, we propose an enhanced Random Sample Consensus algorithm with ground point removal (RANSAC-B); the process flow for the RANSAC-B method is listed as follows.

**Figure 2:**
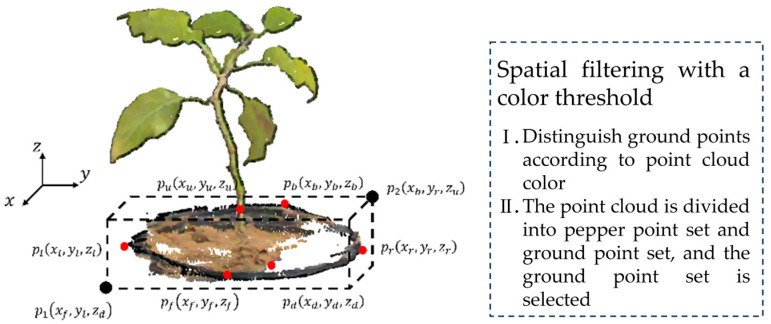
Ground point removal diagram.

Step 1. Spatial filtering. Firstly, the ground points set is distinguished from the original point cloud using the proposed spatial filtering with a color threshold. This step helps to isolate the ground points with colors not associated with the pepper plant, as shown in Figure 2.

Step 2. Bounding box. Secondly, the max boundary six directions, front ($p_{f}$), back ($p_{b}$), left ($p_{l}$), right ($p_{r}$), up ($p_{u}$), and down ($p_{d}$), are identified by employing the maximum sorting algorithm. Based on the six directional boundary points, a bounding box can be formed with all of the ground points enclosed.

Step 3. Key vertices. Thirdly, the key vertices of the identified bounding box are the two diagonal vertices that represent its geometric parameters—$p_{1}\left(x_{max},y_{min},z_{min}\right)$ and $p_{2}\left(x_{min},y_{max},z_{max}\right)$, for example.

Step 4. Ground point removal. Finally, the RANSAC algorithm is employed to remove the invalid ground points within the identified bounding box.

The RANSAC algorithm is applied as follows:

Let Ω represent the point cloud space of the background frame, and let all points within Ω constitute the point set $P_{R}$. We randomly select the first group of three sample points $p_{1},p_{2},p_{3}\in P_{R}$. Subsequently, we determine the plane equation formed by $p_{1},p_{2},p_{3}$ as $A_{i}x+B_{i}y+C_{i}z+D_{i}=0$. Next, we substitute the remaining points from point set $P_{R}$ (excluding the sampled points) into the plane equation, and calculate the distance $d_{R}$ from each point to the plane using Equation (1). Following this, we compare $d_{R}$ with the distance threshold $d_{t_{R}}$: if $d_{R}<d_{t_{R}}$, the point is considered an inlier; if $d_{R}>d_{t_{R}}$, the point is considered an outlier. This process is repeated, resulting in multiple sets of inlier and outlier points. The set with the highest number of inlier points is designated as the optimal point set. Based on the optimal point set, we obtain the optimal plane equation and filter out points in the point cloud. To determine an appropriate distance threshold $d_{t_{R}}$, we assess its impact on the fitting performance. By gradually increasing or decreasing the threshold, we aim to find a threshold that effectively distinguishes ground points.

(1) $$d_{R}=\frac{A_{i}x_{0}+B_{i}y_{0}+C_{i}z_{0}+D_{i}}{\sqrt{{A_{i}}^{2}+{B_{i}}^{2}+{C_{i}}^{2}}}.$$

#### 2.3.2. K-Means Based Segmentation

As for the segmentation of the pepper leaves from its point cloud, the K-means clustering method is employed. The steps and flowchart are depicted as follows.

**Figure 3:**
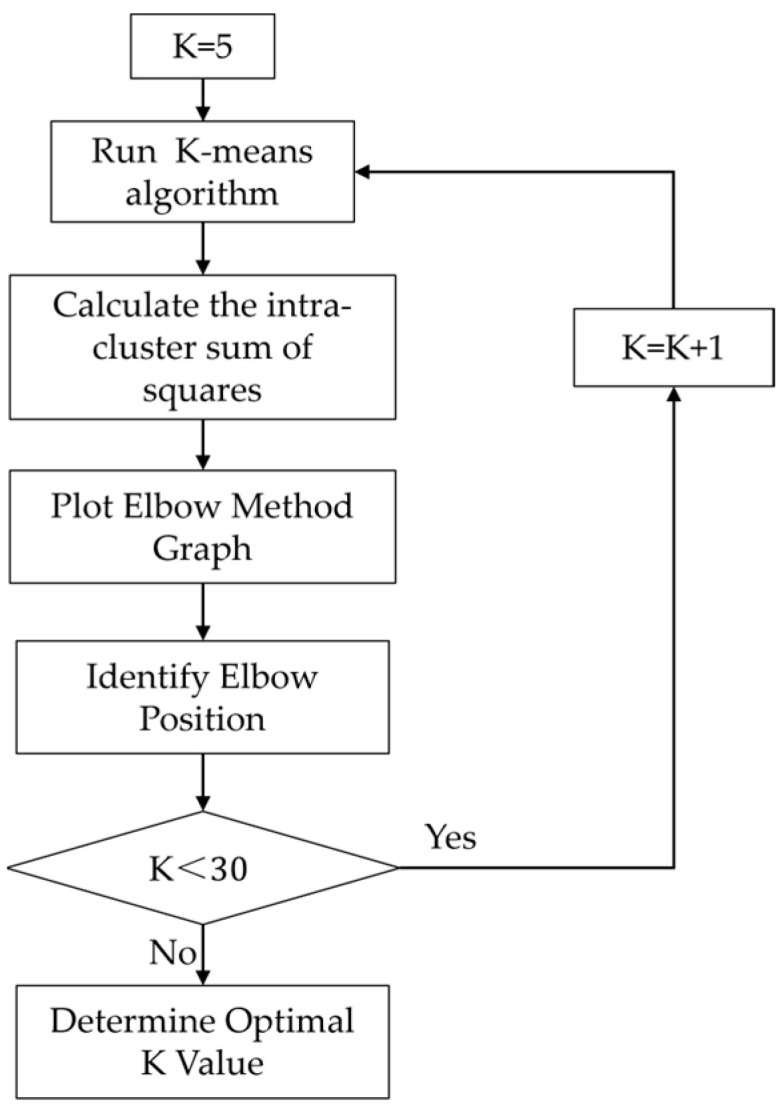
Flowchart of the elbow method.

Step 1. Use the elbow method to determine the optimal value of K as the number of segments for clustering segmentation [24]. Choose a range for K, run the K-means for each value, calculate aggregation metrics, and identify the optimal K from the elbow method graph (see Figure 3).

Step 2. Initialization. For the determined optimal K, initialize the clustering centers ($d_{k0}$) using a random algorithm.

Step 3. Object Assignment. Assign each point cloud object to its nearest clustering center.

Step 4. Compute New Centroids. Calculate new centroid positions (d_k_) by averaging the points assigned to each cluster.

Step 5. Iterative Computation. Repeat Steps 3 and 4 iteratively until the convergence criteria ($d_{k}-d_{k0}<\theta$) are met.

Step 6. Algorithm Termination. If the convergence criteria are not met, continue iterating; otherwise, terminate the algorithm and output the final clustering results.

The initial cluster number is set to 5; the maximum cluster number is set to 30. The elbow method graph is constructed by plotting the within-cluster sum of squares for each K value. The horizontal axis represents the range of K values, and the vertical axis denotes the corresponding within-cluster sum of squares. The elbow position on the graph is identified as the point where the sharp decline in the aggregation metric transitions to a more gradual decrease. The K value associated with this elbow position is considered the optimal initial K value for our clustering analysis.

#### 2.3.3. MLS-Based Point Cloud Smoothing

**Figure 4:**
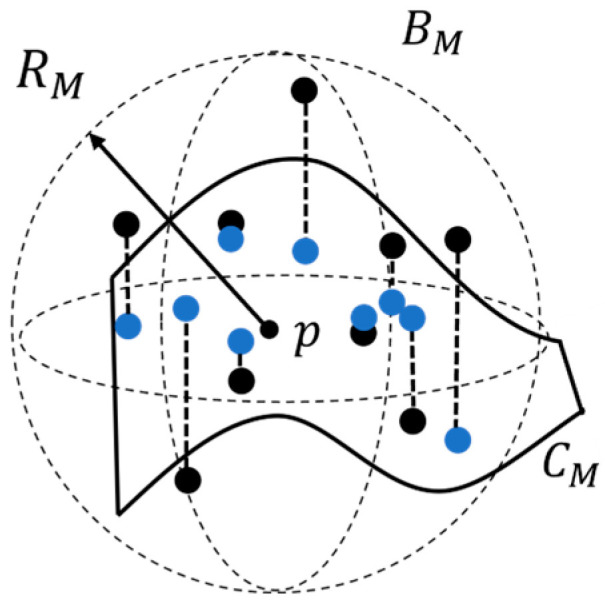
Overview of the MLS algorithm.

The MLS method is employed for point cloud smoothing, and the specific process can be summarized as follows (see Figure 4):

Step 1. Randomly select a point p from the pepper leaf point cloud.

Step 2. Define a spherical region $B_{M}$ with a radius $R_{M}$ centered at point p.

Step 3. Collect all points within the spherical region $B_{M}$ to form the point set $P_{M}$.

Step 4. Use the Least Squares Surface Fitting method to fit a surface, $C_{M}$, to all points in $P_{M}$.

Step 5. Divide the point set $P_{M}$ into two groups: inliers (points within the fitted surface $C_{M}$, depicted in blue) and outliers (points outside the fitted surface $C_{M}$, depicted in black).

Step 6. Project all outliers from $C_{M}$ onto the surface of $C_{M}$ using the normal projection method, thereby smoothing the pepper leaf point cloud.

### 2.4. Euclidean-Distance-Based Phenotypic Parameter Measurement

#### 2.4.1. Leaf Length

(1) Manual leaf length measurement

The leaf is extended and a vernier caliper with a precision of 0.1 mm is employed to measure the length from the leaf tip to the leaf base, denoted as leaf length $l_{a}$.

(2) Euclidean-distance-based leaf length measurement

**Figure 5:**
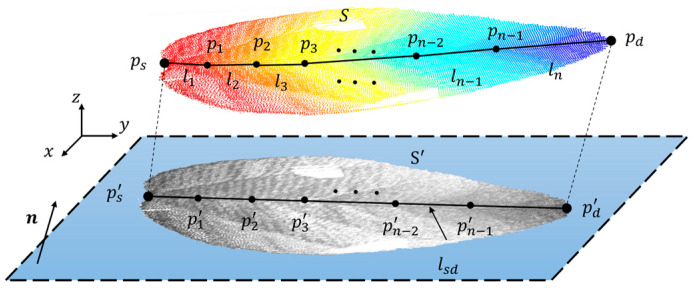
Schematic diagram of the calculation of leaf length.

As depicted in Figure 5, the spatial point cloud surface of the leaf is set as S. Then, S is projected onto the horizontal plane in space, denoted as the xy plane, to obtain $S^{\prime }$, and the starting point $p_{s}^{\prime }$ is assigned at the leaf base and the ending point $p_{d}^{\prime }$ at the front end of the leaf in $S^{\prime }$. Next, $p_{s}^{\prime }$ and $p_{d}^{\prime }$ are connected to form a straight-line segment $l_{sd}$. Subsequently, $l_{sd}$ is divided into n equal segments, with the i-th segment point $p_{i}^{\prime }$. After that, the normal vector $\vec{n}$ is computed, proceeding from $p_{s}^{\prime }$ along $l_{sd}$ in the direction perpendicular to $\vec{n}$, and the corresponding projection points $p_{i}$ are located within S for $p_{i}^{\prime }$. Sequentially $p_{s}$,$p_{1}$,……$,p_{n-1}$ and $p_{d}$ are connected to form line segments $l_{1},l_{2},$……$l_{n}$. Finally, the real leaf length $l_{p}$ can be calculated using Equation (2).

(2) $$l_{p}=\mu_{1}\sum_{i=1}^{n_{1}}l_{i}$$

where $l_{i}$ is the estimated leaf length, $\mu_{1}$ is the length scale, and $n_{1}$ is the number of segments.

#### 2.4.2. Leaf Perimeter

(1) Manual leaf perimeter measurement

Firstly, the obtained images are binarized, which are taken by laying the pepper leaf flat 30–50 cm below the camera. Secondly, the edge point of each binarized image is detected, and the pixel distance $C_{i}$ between adjacent points $p_{i}$ and $p_{i+1}$ is calculated. Thirdly, the perimeter measurement $C_{a}$ is performed.

(3) $$C_{a}=\mu_{2}\sum_{i=1}^{n_{2}}C_{i}\;$$

where $\mu_{2}$ is the pixel length scale and $n_{2}$ is the total number of the edge points of the pepper leaf.

(2) Euclidean-distance-based leaf perimeter measurement

**Figure 6:**
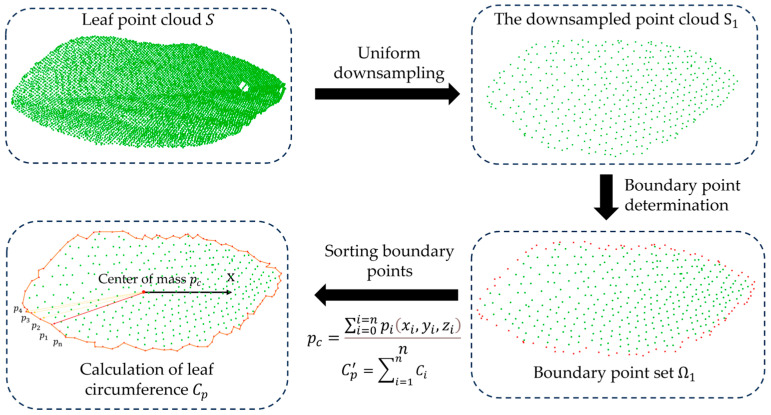
Overview of leaf perimeter acquisition.

In order to lessen the consumption of computing resources, uniform downsampling [25] is used to downsample the original point cloud S. The downsampled point cloud is labeled as $S_{1}$ and is subsequently utilized to apply an edge extraction algorithm [26], which is used to identify the boundary point set $\Omega_{1}$ (see Figure 6).

Next, the Euclidean distance $C_{pi}$ between adjacent points in a clockwise direction along the set $\Omega_{1}$ is calculated. Finally, the real leaf perimeter $C_{p}$ can be calculated using Equation (4).

(4) $$C_{p}=\mu_{1}\sum_{i=1}^{n_{3}}C_{pi}$$

where $n_{3}$ is the number of boundary point line segments.

#### 2.4.3. Leaf Area

(1) Manual leaf area measurement

Firstly, a two-dimensional image of the leaf is captured from a precise position, 30 cm directly above the leaf’s central point. Subsequently, the image is binarized. Then, the black pixels representing the leaf area are accurately counted and used to calculate the area measurement $S_{a}$.

(5) $$S_{a}=\mu_{3}\sum_{i=1}^{n_{4}}S_{i}$$

where $\mu_{3}$ is the pixel area scale and $n_{4}$ is the total number of leaf pixels.

(2) Euclidean-distance-based leaf area measurement

**Figure 7:**
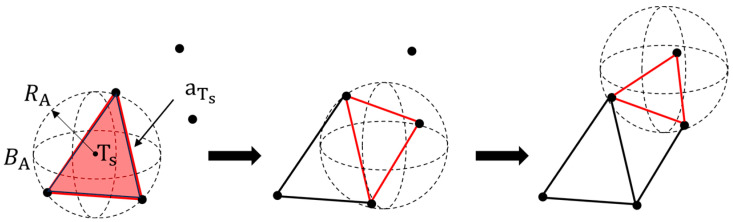
Diagram of the triangulation.

For pepper leaf area calculation, surface reconstruction is performed using the point cloud, employing the Ball Pivoting algorithm [27] for triangular meshing. A starting triangle is randomly selected as the starting triangle $T_{s}$. A sphere $B_{A}$ with a radius $R_{A}$ is defined, which contacts the three vertices of $T_{s}$. Starting from any edge $a_{T_{s}}$ of the starting triangle $T_{s}$, the sphere $B_{A}$ is rotated in space. When $B_{A}$ encounters a new point, a new triangle is created by connecting the edge $a_{T_{s}}$ with the new point. This process is repeated until all points in the point cloud have been visited, as illustrated in Figure 7.

**Figure 8:**
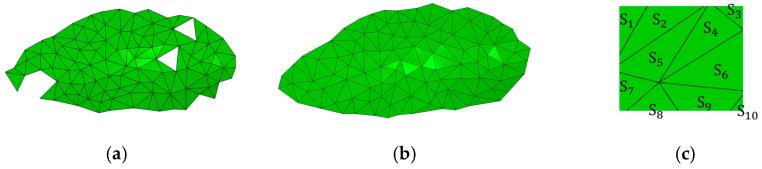
Triangle reconstruction results. (**a**) $R_{A}$ = 3; (**b**) $R_{A}$ = 2~5; (**c**) triangulated local.

In this method, defining the sphere radius $R_{A}$ is a crucial step in surface reconstruction. If the $R_{A}$ is too small, the rolling sphere may pass through gaps in the point cloud without finding new points, resulting in holes in the reconstructed surface, as depicted in Figure 8a. To address this issue and reduce the occurrence of holes during reconstruction, the number of input rolling spheres was increased to 4, with radii ranging from 2 to 5. During the reconstruction process, smaller spheres were employed in dense point cloud regions, while larger spheres were used in sparse point cloud areas for triangulating the surface. The results are illustrated in Figure 8b, demonstrating a more complete reconstruction compared to the situation depicted in Figure 8a.

In Figure 8c, local triangulation is performed. The area of each triangle is calculated based on its vertices, denoted as $S_{i}$. The sum surface area $S_{p}$ is calculated using Equation (6).

(6) $$S_{p}=\mu_{4}\sum_{i=1}^{n_{5}}S_{i}$$

where $\mu_{4}$ is the point cloud area scale and $n_{5}$ is the total number of triangular meshing.

## 3. Results and Discussion

**Figure 9:**
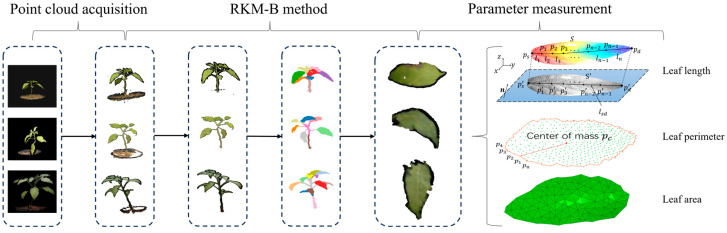
Overview of the procedure.

In accordance with the previously outlined methodology, the comprehensive procedure is depicted in Figure 9.

### 3.1. Point Cloud Acquisition

**Figure 10:**
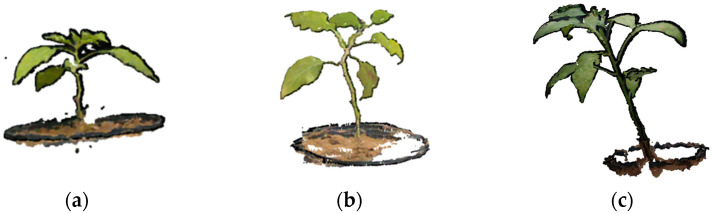
The preprocessed point cloud obtained at different periods. (**a**) The 14th day; (**b**) the 28th day; (**c**) the 42nd day.

As described in Section 2.2, the images were captured using a stereo camera, followed by filtering and stereo matching to obtain the original point cloud. Subsequently, the point clouds preprocessed at different periods, as illustrated in Figure 10, and underwent ICP registration [23] and uniform downsampling [25] with a sampling radius of 20. The respective point cloud numbers were 53,487, 47,356, and 125,690.

### 3.2. RKM-B-Based Point Cloud Processing

**Figure 11:**
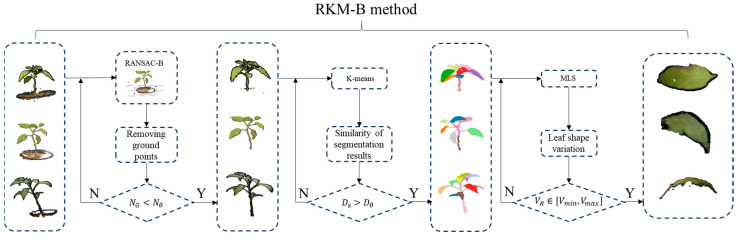
Overview of the RKM-B method procedure.

The point cloud preprocessing method, which incorporates the improved Random Sample Consensus (RANSAC-B), K-means, and Moving Least Squares techniques, is collectively referred to as RKM-B, in contrast to the unmodified RKM method. Comprehensive processing using the RKM-B method is illustrated in Figure 11.

In the ground point removal stage, $N_{G}$ represents the total number of point clouds in the background box, while $N_{\theta }$ denotes the number of stem point clouds in the ground box and is calculated using Equation (7).

(7) $$N_{\theta }=\pi r^{2}h\rho_{point}$$

where r is the stem radius, h is the background box height, and $\rho_{point}$ is the number of stem point clouds per unit volume.

In the leaf segmentation stage, the similarity coefficient $D_{s}$ (Equation (8)) is introduced to compare the similarity between clustered segmentation results and actual leaves [28]. A segmentation threshold $D_{\theta }$ is determined based on empirical considerations, and for leaf quantities ranging from 0 to 10, the coefficient $D_{\theta }$ is assigned a value of 0.88. In the case of leaf quantities between 11 and 25, $D_{\theta }$ takes a value of 0.84. Furthermore, when the leaf quantity surpasses 25, $D_{\theta }$ is set to 0.80.

(8) $$D_{s}=\frac{2\times \left(L_{K}\cap L_{R}\right)}{L_{K}+L_{R}}$$

where $L_{K}$ is the number of point clouds identified and $L_{R}$ is the number of point clouds in actual leaves.

Leaf smoothing is determined based on the volume ratio $V_{R}$ of the minimum bounding box of the leaf point cloud. The maximum volume ratio $V_{max}$ is set at 0.9, while the minimum value $V_{min}$ is designated as 0.7.

**Figure 12:**
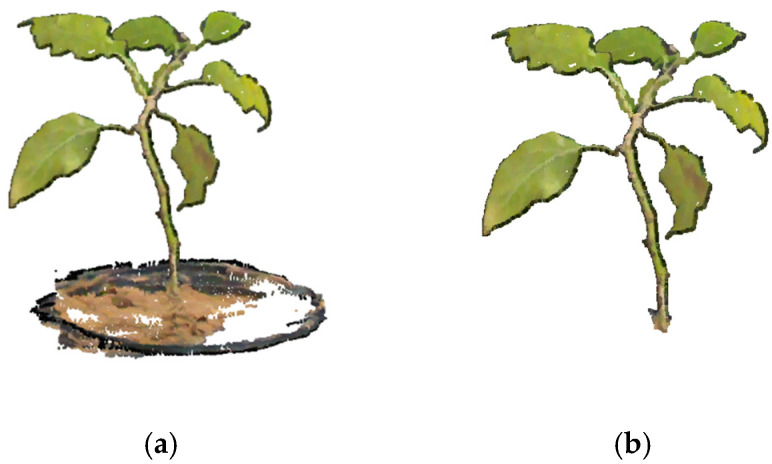
RANSAC-B ground point filtering. (**a**) Before processing; (**b**) after processing.

Utilizing the RANSAC-B algorithm introduced in Section 2.3.1, the results are illustrated in Figure 12.

**Figure 13:**
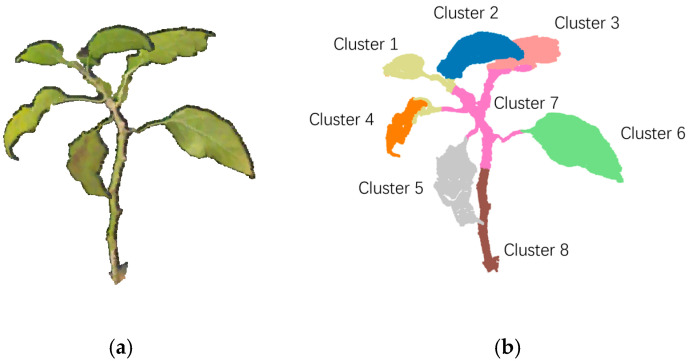
Segmentation of pepper leaves. (**a**) Point cloud of pepper; (**b**) point cloud clustering results.

Continuing from ground point removal, leaf segmentation is implemented using the method outlined in Section 2.3.2, as depicted in Figure 13. In Figure 13b, the point cloud is segmented into eight clusters, each representing an individual leaf, while the stem is divided into two clusters.

**Figure 14:**
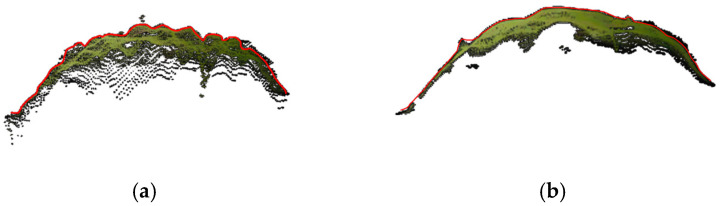
Schematic diagram of leaf smoothing treatment. (**a**) Original point cloud; (**b**) post-smoothed point cloud.

Following leaf segmentation, individual leaves display fluctuation errors and uneven contours in the side view of the point cloud, indicating significant undulations. The outcomes, post-application of the smoothing method described in Section 2.3.3, are illustrated in Figure 14.

The smoothness $\rho_{i}$ of a single leaf is defined according to [29] as Equation (9). An average angle of 30° corresponds to a smoothness value of 0.033, while a 45° angle corresponds to a smoothness value of 0.022. The larger the smoothness value, the smaller the average angle.

(9) $$\rho_{i}=\frac{1}{\frac{1}{m}\sum_{a=1}^{m}\left(\frac{1}{20}\sum_{b=1}^{20}\varphi \left(a,b\right)\right)}$$

where i denotes the i-th leaf, $\varphi \left(a,b\right)$ represents the angle between the normal vectors of point a and its neighboring 20 points b, and m is the total number of points in the point cloud.

The average smoothness of each leaf is computed using Equation (10).

(10) $$\bar{\rho }=\sum_{i=1}^{n}\frac{1}{n}\rho_{i}$$

where n is the total number of leaves at a specific period.

Considering the results of the aforementioned processing, a comparative analysis was conducted, examining the RKM-B processing method and the untreated dataset concerning differences in the point cloud number, leaf smoothness, algorithmic execution time, and other aspects. Table 1 succinctly presents the comprehensive results of this comparative assessment.

From Table 1, it can be seen that the total time consumption as well as “R” and “K” are significantly reduced in the three periods. However, for “M”, the changes are gentle, and in some instances, there is even a time increase, particularly on the 42nd day. This is because ground point removal has no impact on the quantity and morphological features of leaf point clouds, resulting in no sharp change in the time consumption for “K.” Contrarily, for “R,” the time gradually increases with the growth of crops. This is due to the easier differentiation between ground points and stems as the leaves expand, leading to a reduction in interference. However, for “K,” the reduction in time diminishes as the crops grow. This is due to the increase in the number of points involved in segmentation as the peppers grow, leading to a decrease in the reduction in processing time. The total reduction in time stabilizes at around 2 s, but the baseline processing time continues to increase with crop growth.

From the perspective of smoothness, the increases in smoothness for the leaves at the three growth periods are 0.003, 0.004, and 0.002, corresponding to average angle reductions of 2.7°, 6.1°, and 3.5°, respectively. Compared to the untreated smoothness, both “RKM-B” and “RKM” exhibit significant improvements in smoothness. The baseline smoothness gradually decreases across the three growth periods. This outcome is attributed to the relatively smaller size and minimal shape changes in the leaves at the 14th day of growth, resulting in a predominantly flat leaf surface. As the leaves grow, at the 28th day and 42nd day of growth, the leaves become more fully developed, gradually presenting certain curved angles in space.

### 3.3. Leaf Length Measurement

**Figure 15:**
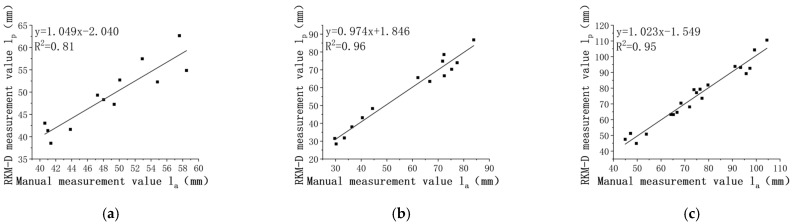
Analysis of leaf length correlation. (**a**) the 14th day; (**b**) the 28th day; (**c**) the 42nd day.

Leaf lengths, denoted as $l_{a}$ and $l_{p}$, are presented separately for the 14th, 28th, and 42nd days in Figure 15. It is evident that $l_{a}$ and $l_{p}$ exhibit a linear relationship.

The R^2^ values for leaf length are all above 0.81 across various pepper growth periods. Moreover, the R^2^ tends to increase with both leaf growth and the number of leaves.

The MAE values for leaf length are 2.57 mm, 3.34 mm, and 3.35 mm, showing a slight increase with leaf growth. After the first measurement period, the MAE stabilizes, with the maximum value not exceeding 3.50 mm.

The RMSE values are 2.90 mm, 3.63 mm, and 3.72 mm, respectively.

Throughout the experiment, the obtained MRE remains less than 5.93%, with MRE values of 5.13%, 5.92%, and 4.65% for different periods.

**Figure 16:**
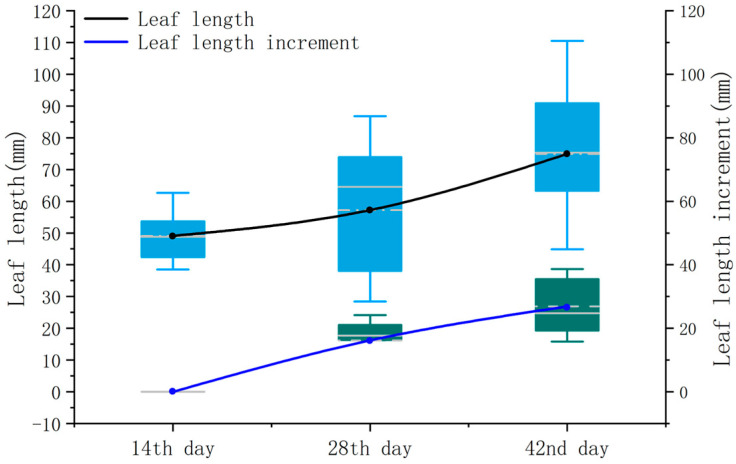
Leaf length versus incremental box plot.

From Figure 16, it can be observed that the leaf length gradually increases over the three periods, with average values of 49.12 mm, 57.24 mm, and 74.94 mm. This trend is attributed to the chosen measurement periods, all of which are in the early stages of pepper development, where leaf growth is rapid and new leaves are continuously generated. By examining the growth increment of leaf length, it was found that the increment in the third period was greater than that in the second period. This phenomenon may be due to an increase in leaf quantity, intensifying the overall physiological activities of the crop and resulting in a higher demand for resources. Consequently, a larger increase in leaf length is observed during the third measurement period.

### 3.4. Leaf Perimeter Measurement

**Figure 17:**
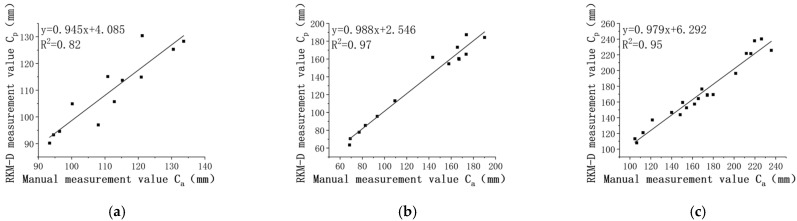
Correlation analysis of leaf perimeter. (**a**) The 14th day; (**b**) the 28th day; (**c**) the 42nd day.

The leaf perimeters, denoted as $C_{a}$ and $C_{p}$, for the three periods are illustrated in Figure 17.

The R^2^ values were 0.82, 0.97, and 0.95, respectively. The lower R^2^ during the first measurement period is attributed to the smaller number of leaves.

The MAE for the pepper leaf perimeter was 5.04 mm, 6.17 mm, and 7.20 mm, indicating an increase in the average absolute error with leaf development, with larger errors observed in the later periods of leaf growth.

The RMSE exhibited a similar trend to the MAE, with values of 5.97 mm, 7.77 mm, and 8.36 mm, respectively.

Throughout the experiments, the MRE remained less than 4.50%, with values of 4.41%, 4.48%, and 4.30%, respectively.

**Figure 18:**
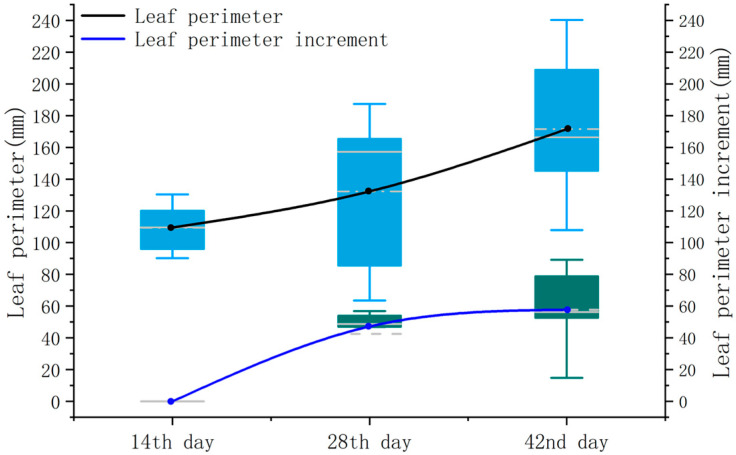
Leaf perimeter versus incremental box plot.

The trend in the leaf perimeter and its increment is generally similar to that of the leaf length. From Figure 18, it can be observed that the average values of the leaf perimeter over the three periods were 109.5 mm, 132.4 mm, and 171.5 mm. It is noteworthy that the leaf perimeter increment on the 42nd day exhibits greater fluctuations compared to leaf length. This could be attributed to different parts experiencing varying growth rates, with the growth rate at the leaf margin potentially influenced by local environmental conditions, nutrient distribution, or genetic regulation, leading to uneven leaf perimeter growth.

### 3.5. Leaf Area Measurement

**Figure 19:**
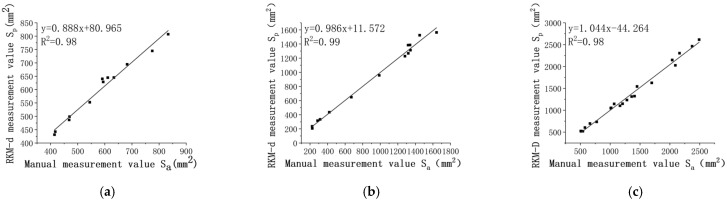
Correlation analysis of leaf area across three different growth periods. (**a**) The 14th day; (**b**) the 28th day; (**c**) the 42nd day.

Linear regression analyses were conducted using manual measurement values $S_{a}$ and RKM-D measured values $S_{p}$, as depicted in Figure 19. The results presented are for three measurement periods.

The R^2^ values were 0.98, 0.99, and 0.99, all surpassing 0.97. This indicates a significant correlation between the calculated values from the pepper area point cloud and manual measurements.

The MAE for the pepper leaf area was 24.59 mm^2^, 39.35 mm^2^, and 64.65 mm^2^, respectively. The MAE increases with leaf development, with larger absolute errors observed in the later periods of leaf growth. The first measurement period exhibits the smallest average absolute error in leaf area, while the third measurement period shows the largest, reaching a maximum value of 64.65 mm^2^.

The RMSE follows a trend similar to the MAE, with values of 27.07 mm^2^, 44.94 mm^2^, and 73.05 mm^2^, respectively.

Throughout the experiments, the MRE remained less than 4.96%, with small variations, at 4.33%, 4.95%, and 4.76%, respectively.

**Figure 20:**
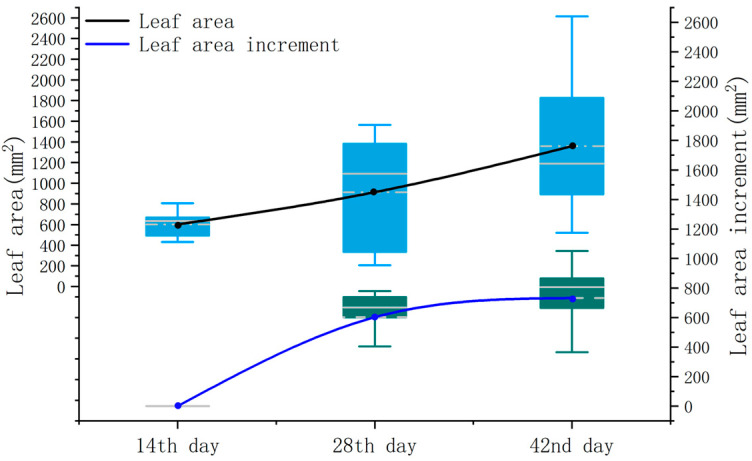
Leaf area versus incremental box plot.

The overall trend in the leaf area and its increment is similar to the leaf length and perimeter. Over the three periods, the average values of the leaf area gradually increase (see Figure 20), measuring 601.4 mm^2^, 914.5 mm^2^, and 1358.6 mm^2^, respectively. However, unlike the leaf length and perimeter, the measurement of the leaf area shows a relatively higher correlation coefficient during the first measurement period. This is primarily because, when dealing with irregularly shaped leaves, the leaf area, as a comprehensive measure of the overall leaf size, can mitigate the measurement inaccuracies caused by local errors, thus enhancing the overall measurement correlation.

## 4. Conclusions

This paper presents a novel method for measuring crop leaf phenotypic parameters, named RKM-D. Using peppers as the representative research subject, manual measurements and RKM-D point cloud measurements were conducted on the 14th, 28th, and 42nd days of pepper growth. The results demonstrate a strong linear relationship between the proposed RKM-D method and the ground truth, with minimal parameter errors.

The RKM-D method includes preprocessing steps such as ground point removal using RANSAC-B, leaf segmentation using K-means, and leaf smoothing using MLS. Additionally, the method incorporates a phenotype measurement approach for the pepper leaf length, perimeter, and area based on Euclidean distance.

Specifically, for leaf length measurements, the R^2^ values exceed 0.81, the MAE is less than 3.50 mm, the MRE is less than 5.93%, and the RMSE is less than 3.92 mm. For the leaf perimeter, the R^2^ exceeds 0.82, the MAE is less than 9.10 mm, the MRE is less than 4.50%, and the RMSE is less than 11.27 mm. Leaf area measurements show R^2^ values exceeding 0.98, an MAE less than 85.58 mm^2^, an MRE less than 4.96%, and an RMSE less than 3.92 mm^2^.

This method proves effective in achieving accurate phenotypic measurements of pepper leaves, providing valuable insights for research on pepper leaf phenotypic parameters.

Furthermore, future research will focus on three main aspects: extending leaf measurements across multiple time points to establish growth curves, investigating environmental factors’ impact on plant growth for accurate assessments, and implementing advanced agricultural intelligence techniques for automated and intelligent plant monitoring. Through these efforts, we aim to comprehensively understand plant growth dynamics and contribute to the advancement of sustainable agriculture.

## Figures and Tables

**Table 1:** Comparison of the effects of methods in each period.

Period		Time Consuming (s)				$\mathrm{Smoothness}\;\bar{\rho }$
		R	K	M	Total	
14th day	RKM	1.58	1.41	0.52	3.51	0.032
	RKM-B	0.96	0.39	0.49	1.84	0.035
	Original	/	/	/	/	0.029
28th day	RKM	1.73	1.57	0.43	3.73	0.024
	RKM-B	0.97	0.56	0.41	1.62	0.028
	Original	/	/	/	/	0.020
42nd day	RKM	2.28	2.52	1.55	6.35	0.022
	RKM-B	0.73	1.79	1.59	4.11	0.024
	Original	/	/	/	/	0.017

Notes: “Smoothness” is the average smoothness of individual leaf segments within each specified interval; “R” is RANSAC-B algorithm and RANSAC algorithm; “K” is K-means algorithm; “M” is MLS algorithm; “total” is the total running time; RKM is a combination of RANSAC, K-means, and MLS algorithms; RKM-B is a combination of RANSAC-B, K-means, and MLS algorithms; “Original” is the smoothness of the leaf without any treatment.

## Data Availability

Data are contained within the article.
